# Dental anxiety and empathy among undergraduate oral health students in Norway, South Africa and Namibia

**DOI:** 10.2340/aos.v84.43424

**Published:** 2025-04-09

**Authors:** Faheema Kimmie-Dhansay, Ann Catrin Høyvik, Ahmed Bhayat, Faezah Hussain, Arannikah Karunakaran, Besime Kaya, Pagollang Motloba, Cathrine Malau, John Rutabanzibwa, Shenuka Singh, Vivienne Wilson, Ingvild Johnsen Brusevold

**Affiliations:** a Department of Community Dentistry, Faculty of Dentistry, University of the Western Cape, Cape Town, South Africa; b Department of Pediatric Dentistry, Behavioral Science and Forensic Dentistry, University of Oslo, Oslo, Norway; c Department of Community Dentistry, School of Dentistry, University of Pretoria, Pretoria, South Africa; d Department of Community Dentistry, Sefako Makgatho Health Sciences University, Ga-Rankuwa, South Africa; e Department of Community Dentistry, School of Oral Health Sciences, University of the Witwatersrand, Johannesburg, South Africa; f Department of Community Dentistry and Orthodontics, Windhoek, Namibia; g School of Health Sciences, Discipline of Dentistry, University of the KwazZulu Natal, Durban, South Africa

**Keywords:** empathy, dental anxiety, oral health students

## Abstract

**Purpose/objectives:**

Dental anxiety is a common type of fear that can complicate dental treatment. The dental practitioner is crucial in both treating dental fear and anxiety as well as prevent it from arising. The ability to feel empathy is important in that matter. The dental practitioner’s own level of dental anxiety can possibly affect his or her ability to treat patients in an empathetic manner. The aim of this study was to assess and examine the relationship between level of empathy and dental anxiety in undergraduate oral healthcare students from Namibia, South Africa and Norway.

**Material and methods:**

A cross-sectional study was performed. Questionnaires were distributed, and responses were analyzed anonymously. Dental anxiety was assessed using Modified Dental Anxiety Scale (MDAS), and empathy level was assessed using Toronto Empathy Questionnaire (TEQ). Data were presented as means or medians and analyzed using a linear regression model in STATA with a 5% level of significance.

**Results:**

The response rate was 16.0%, and 298 completed questionnaires were received. MDAS was low in all groups (medians 7–10), however, significantly lower in Norway compared to Namibia and South Africa. The mean TEQ score was 46.8 in Namibia, 47.5 in South Africa and 50.4 in Norway, all above average empathy levels but significantly higher in Norway than in Namibia and South Africa.

**Conclusions:**

Oral healthcare students in Africa and Norway showed high empathy and low dental anxiety, which is reassuring for future oral health care professionals.

## Introduction

Dental anxiety is originally defined as ‘the patient’s response to the stress specific to the dental situation’ [1] and may explain avoidance of dental care [2–4], resulting in neglect of personal oral health [5]. Many theories exist that explicate dental anxiety. The theory of classic conditioning describes how anxiety may be a consequence to negative or traumatic prior experience [2, 4, 6]. Other theories such as social learning, biological influences, cognitive factors, personality, and pain-related factors also exist to explain the theory of dental anxiety [7, 8].

High levels of dental anxiety result in adverse reactions toward dental therapy [8, 9] and result in patients with poorer oral health-related quality of life (OHRQoL). Poorer OHRQoL scores were seen to be positively associated with an increase in dental anxiety scores [10]. Dental anxiety may also hinder the proper diagnosis of the main complaint of patients. In addition, patients with higher dental anxiety had more missing teeth [3].

Certain dental procedures exhibited higher dental anxiety such as dental surgery followed by root canal treatment and tooth drilling compared to pain alone [4, 11]. Female dental students tend to have a higher dental anxiety than their male counterparts [12]. Dental students early in their training display higher levels of dental anxiety compared to dental students who were closer to graduation [12]. Furthermore, older patients had lower levels of anxiety compared to patients who were younger in age [13].

Empathy is the ability to envision someone else’s suffering and feel compassion for what they are going through. Although intuitive, some people may be indifferent toward another person in pain, which is an indication that empathy is not inherently built into a person. Kraft-Todd et al. defined empathy as ‘a social-emotional ability having two distinct components: one affective: the ability to share the emotions of others, and one cognitive: the ability to understand the emotions of others’ [14].

Signs of empathy are being a good listener, being able to detect another person’s feelings, trying to help other people who are suffering, caring deeply about other people, and finding difficulty in setting boundaries in the relationships with other people. However, having empathy also means that you can become overstimulated, overwhelmed, and prone to burnout as you always think of other people first [15].

Studies have shown that empathy is important in the relationship between the dentist and the patient [16]. Furthermore, a recent study among dental students and educators in France showed that their level of empathy was high [17]. Dental practitioner empathy has been positively correlated with an increased patient compliance in orthodontics [18], lowered patient anxiety [19, 20], and increased patient satisfaction [20].

From other health care workers, empathy has proven to affect patients’ health, e.g. it has been shown that physicians who display empathy during communication with patients resulted in a higher proportion of patients with better glycemic control and lower low-density lipoprotein cholesterol (LDL-C) [21]. Patients who were treated with empathy also showed a shorter viral-cold duration and a greater amount of change in interleukin-8 and neutrophil levels [22]. In addition, empathetic doctors had a suppressive effect on anxiety in their patients [23] and an increased effect on patient satisfaction [24].

Even though there is translational evidence supporting empathy in dentistry toward patients, there has been evidence to show that empathy is declining in dental students, especially as they advance through their dental education [25, 26]. However, Naguib et al. found no statistically significant difference in empathy scores for different years of training [27]. Furthermore, overall empathy was also determined to be higher in females compared to males [28, 29].

Being empathetic has many other benefits as well, such as being able to socially connect with others by understanding what others are saying, thinking, or feeling and emotional regulation, which means to control one’s own emotions in times of stress [7, 15]. Empathy also promotes helping behaviors; the empath would be inclined to helping others when they experience empathy [7, 15]. There are certain factors that affect empathy, such as how the other person is perceived, past experiences, expectations, and genetics [15, 30].

To the knowledge of the authors, no study has assessed the relationship between dental anxiety and empathy in undergraduate oral healthcare students.

The objectives of this study were

1. To determine the empathy and dental anxiety levels of oral healthcare students at all the Dental Faculties in South Africa, Namibia, and Norway
2. To explore the relationship between empathy and dental anxiety of oral healthcare students at all the Dental Faculties in South Africa, Namibia, and Norway

## Materials and methods

### Study design

A cross-sectional study design was employed. An online questionnaire was designed to determine the dental empathy and dental anxiety using REDcap ® and/or paper in Namibia and South Africa and in Nettskjema in Norway. In Norway, a link and a QR code to the questionnaire were distributed by e-mail to first- and final-year students only, in Namibia to final-year students, while in South Africa, all oral healthcare students received a link to the questionnaire by e-mail. In Norway, a reminder was sent twice via e-mail to the students at the University of Oslo (UiO), University of Bergen (UiB), and UiT The Arctic University of Norway (UiT). In addition, three of the authors gave oral reminders to the students in Oslo once during the lecture. It was not possible to reach out in person to the students in UiB and UiT. A paper form was not used in Norway as only digital responses allowed the e-mail addresses to be collected and stored in safe storage, only accessible by the project administrator. This enables a later follow-up study.

One reminder was sent to the students at University of the Witwatersrand (WITS) (paper form only), two reminders were sent by e-mail to the students at University of the Western Cape (UWC), University of the KwaZulu Natal (UKZN), Sefako Makgatho Health Sciences University (SMU) and University of Namibia (UNAM), and three reminders were sent by e-mail, Whatsapp, and personal invitation to the students at University of Pretoria (UP).

### Inclusion criteria

All undergraduate oral healthcare students registered in 2022 from the Dental Faculties/Schools of the University of the Western Cape, University of Pretoria, University of the Witwatersrand, Sefako Makgatho Health Sciences University, and University of the KwaZulu Natal. At the University of Namibia, only final-year students were invited. In Norway, all first- and final-year dental students from UiO, UiT, and UiB were invited.

### Exclusion criteria

Qualified dentists and postgraduate students were excluded from this study.

### Questionnaires

Dental anxiety was assessed utilizing the Modified Dental Anxiety Scale (MDAS) tool that consists of 5 specific questions about dental anxiety collected as Likert-type data, where 1 equates to ‘Not anxious’ and 5 equates to ‘Extremely anxious’ [31]. The MDAS score is calculated by summating the answers to the five questions, and the maximum score allotted is indicative of a higher anxiety level. A cut-off of 19 indicates a highly dentally anxious subject, possibly dentally phobic subject. The MDAS tool was previously translated to and validated for use in Norway; thus the Norwegian version was used in Norway [32].

Empathy in oral health students was collected using the Toronto Empathy Questionnaire (TEQ), which has been used extensively in determining the dental empathy levels of undergraduate dental students, globally [33]. The TEQ consists of 16 questions regarding how the subject feels when another person is displaying happiness or sadness and is rated on a Likert scale from 0 (which is ‘Never’) to 4 (which is ‘Always’). A higher score indicates a higher level of empathy. According to Spreng et al. [33] who developed the TEQ, males had average scores between 44.63 and 44.46, while females had average scores between 44.62 and 48.93; thus the gender differences were considered moderate. In the present study, for use among Norwegian students, the questionnaire was translated to Norwegian according to standards described by Chang et al. [34]. First, a translation was performed independently by three of the authors: FH, AK, and BK. Each of the three Norwegian translations was then back-translated to English by three independent persons having English as first language and who were fluent in Norwegian. The authors FH, AK, BK, ACH, and IJB together chose the Norwegian translation that gave back-translation that was most similar to the original English version. The questionnaire was then tested by the authors. In South Africa and Namibia, the original form in English was used, as English is the teaching language in both countries. No further cultural adaptation was considered necessary.

Additional background questions on demographics and dental visits were included in the questionnaire as shown in Table 1.

**Table 1:** Demographics, MDAS and TEQ scores, and background questions.

Factor	Level	Total	Norway	South Africa	Namibia	*P*
Invited students		1,867	276	1,519	72	
Number of participants (% of participants per country)		298	95 (31.9%)	165 (55.4%)	38 (12.8%)	
MDAS_tot, mean (SD)		9.51 (3.8)	7.3 (2.1)	10.1 (3.7)	11.8 (4.6)	< 0.001
MDAS_tot, median (IQR)		8 (6–12)	7.0 (6.0, 8.0)	9.0 (7.0, 12.0)	10.0 (8.0, 14.0)	< 0.001
TEQ_tot, mean (SD)		46.49 (11.2)	50.4 (5.2)	47.5 (6.7)	46.8 (6.8)	< 0.001
TEQ_tot, median (IQR)		49 (43.5–52.3)	51.0 (48.0, 54.0)	48.5 (43.0, 52.0)	48.5 (43.5, 51.0)	< 0.001
What is your sex?	Female	247 (77.7)	74 (77.9%)	138 (79.3%)	25 (67.6%)	0.3
	Male	71 (22.3)	21 (22.1%)	36 (20.7%)	12 (32.4%)	
Did you grow up in an Urban/Rural area	Urban	205 (64.5)	51 (53.7%)	123 (70.7%)	23 (62.2%)	0.02
	Rural	113 (35.5)	44 (46.3%)	51 (29.3%)	14 (37.8%)	
How would you rate your own oral health?	Poor	5 (1.6)	0 (0.0%)	4 (2.3%)	1 (2.7%)	< 0.001
	Fair	38 (12.0)	1 (1.1%)	28 (16.1%)	7 (18.9%)	
	Good	125 (39.3)	33 (34.7%)	69 (39.7%)	18 (48.6%)	
	Very Good	125 (39.3)	47 (49.5%)	67 (38.5%)	8 (21.6%)	
	Excellent	25 (7.9)	14 (14.7%)	6 (3.4%)	3 (8.1%)	
Have you had any negative experiences related to dental care?	No	192 (60.4)	65 (68.4%)	101 (58.0%)	17 (45.9%)	0.047
	Yes	126 (39.6)	30 (31.6%)	73 (42.0%)	20 (54.1%)	
Is there anyone in your nearest family (parents, siblings) who is fearful of dental treatment?	Yes	160 (50.3)	25 (26.3%)	115 (62.2%)	20 (52.6%)	<0.001
	No	158 (49.7)	70 (73.7%)	70 (37.8%)	18 (47.4%)	
What proportion of the population do you think has dental anxiety?	5–20%	33 (10.4)	23 (24.2%)	9 (4.9%)	1 (2.6%)	< 0.001
	20–30%	59 (18.6)	31 (32.6%)	18 (9.7%)	10 (26.3%)	
	30–50%	100 (31.5)	30 (31.6%)	59 (31.9%)	11 (28.9%)	
	> 50%	126 (39.6)	11 (11.6%)	99 (53.5%)	16 (42.1%)	
Dental visits	At least once a year	206 (65.8)	75 (78.9%)	108 (62.1%)	18 (48.6%)	0.001
	Rarely or do not remember	112 (35.2)	20 (21.1%)	66 (37.9%)	19 (51.4%)	
Age Category	< 20 years	59 (18.6)	12 (12.6%)	34 (18.4%)	13 (32.4%)	< 0.001
	20–25 years	216 (67.9)	56 (58.9%)	136 (73.5%)	24 (63.2%)	
	≥ 26	43 (13.5)	27 (28.4) (62.8%)	15 (34.9%)	1 (2.3%)	

MDAS: Modified Dental Anxiety Scale; TEQ: Toronto Empathy Questionnaire; SD: standard deviation; IQR: interquartile range.

### Ethics and human subject issues

Ethical clearance was obtained from participating dental schools (BM22_1_2/SOD 0001/607756) in Namibia and South Africa and in Norway from the Norwegian Data Protection Services (Reference number 607756). The study was conducted according to the Code of Ethics of the World Medical Association (Declaration of Helsinki) and followed the ‘Uniform Requirements for Manuscripts Submitted to Biomedical Journals’ published by the International Committee of Medical Journal Editors (ICJME). Subjects were given an opportunity to consent to participation in the study on the first page of the online survey. Participants were allowed to withdraw from the study at any time.

### Analysis

The data were uploaded into STATA (StataCorp. 2021, Stata Statistical Software: Release 17. College Station, TX: StataCorp LLC). All continuous data were displayed as mean and standard deviation if normally distributed or median and interquartile ranges, otherwise. The final model was analyzed using a linear regression model. During model building, the level of significance for covariates was at 10%. All tests were deemed statistically significant at the 5% level of significance.

### Timeframe

Data collection began in Norway in September 2022 and in April 2023 in South Africa and Namibia. The study concluded in July 2023.

### Budget

This study was self-funded.

## Results

Out of 1,867 students, 298 responded (16.0%) and the majority of them were from South Africa (Table 1). As our primary aim was to study the empathy level among oral healthcare students, we first explored the TEQ score in first- and final-year dental students in Norway. There was no significant difference in the TEQ score between first- and final-year students in Norway; neither was there a difference in MDAS score. We therefore chose to do all the further analyses with all students as one group divided by country. In all countries, there was a higher proportion of female students than male students. There were significantly more students in the age groups older than 25 years in Norway than in South Africa and Namibia. Most of the students rated their own oral health as ‘good’ or ‘very good’. However, in South Africa and Namibia, 16–18% of the students rated their oral health as ‘poor’ or ‘fair’, while only 1% of the students from Norway did so.

### Dental anxiety

The level of dental anxiety as reported by MDAS was low for the majority of students in all countries as the median was between 7 (Norway) and 10 (Namibia) (Table 1). However, the median and mean MDAS score was significantly lower in Norway than in the other countries. The proportion of students that reported having had a negative experience related to dental care was highest in Namibia (54.1%) and lowest in Norway (31.6%). When asked what they thought was the proportion of the population that has dental anxiety, the students from Namibia and South Africa gave a higher estimate than did the students from Norway. Also, there were more students from South Africa (62.1%) and Namibia (54.1%) than from Norway (26.3%) that reported having family members that were fearful (*p* < 0.001).

### Empathy levels

The mean TEQ score showed that students from all three countries had above average empathy levels compared to what was reported by Spreng et al. [33]; however, a significant difference was seen between the countries where students from Norway had the highest score (50.4 vs. 47.5 vs. 46.8 *p* < 0.001) (Table 1).

### Relationship between dental anxiety and dental empathy

Male students had a lower TEQ score than female students, and students who estimated the highest prevalence of dental anxiety in the population had a higher empathy score (Table 2). The empathy levels were not dependent on own dental anxiety (MDAS), frequency of dental visits, urban or rural living, or judgment of their own oral health (Table 2).

**Table 2:** Simple and multiple regression analyses.

TEQ	Simple regression		Multiple regression	
Country	β (95% CI)	*P*	β (95% CI)	*P*
South Africa	-2.87 (-4.44 to -1.3)	< 0.001*	-4.02 (-5.78 to -2.27)	< 0.001*
Namibia	-3.6 (-5.98 to -1.21)	0.003*	-3.86 (-6.39 to -1.33)	0.003
MDAS	-0.15 (-0.34 to 0.05)	0.134	-0.1 (-0.31 to 0.1)	0.307
Urban-Rural				
Rural	0.45 (-1.05 to 1.96)	0.552		
Sex				
Male	-4.06 (-5.73 to -2.4)	< 0.001*	-3.56 (-5.23 to -1.9)	< 0.001*
Dental Visit				
Rarely	-1.06 (-2.57 to 0.46)	0.171		
Oral Health				
Fair	-1.63 (-4.84 to 1.59)	0.32		
Good	-1.6 (-4.71 to 1.52)	0.314		
Very Good	-2.44 (-5.68 to 0.79)	0.138		
Excellent	-2.01 (-7.12 to 3.1)	0.439		
Prevalence of dental anxiety				
20–30%	0.96 (-1.81 to 3.73)	0.495	1.01 (-1.62 to 3.64)	0.452
30–50%	0.86 (-1.7 to 3.43)	0.508	1.67 (-0.87 to 4.2)	0.197
> 50%	2.11 (-0.39 to 4.61)	0.099	3.86 (1.23 to 6.49)	0.004*

MDAS: Modified Dental Anxiety Scale; TEQ: Toronto Empathy Questionnaire.

## Discussion

In this study, the aim was to assess the levels of empathy and dental anxiety in oral heath students in Norway, South Africa, and Namibia to explore relationships between empathy and dental anxiety and finally to determine possible differences between the three countries. Students in Norway reported the lowest dental anxiety scores with MDAS 7. Dental anxiety could be influenced by dental care experience, cultural beliefs, and societal attitudes toward dentistry. In Norway, access to dental care is high compared to South Africa, which could explain the low levels of anxiety compared to Namibia or South Africa, where there is a very low dentist to population ratio. Faculty support, dental education, and peer interactions could have an impact on shaping the anxiety levels toward dental procedures. In Norway, the total burden of caries has decreased to a low level compared to South Africa during the last decades, and that coincides with a reduced level of dental fear and anxiety among 18-year olds as showed in a study from Norway [35]. This is a possible explanation for the lower MDAS in the Norwegian student group. Studies among students in Norway and Jordan found that dental anxiety was lower in dental students than students in other programs [36, 37]. In the study by Storjord et al., dental students had MDAS 8.7, while biology and psychology students had 12.46 and 10.73, respectively, and in Al-Omari et al., dental students had MDAS 11.22, while engineering and medical students had 13.27 and 13.58, respectively [36, 37].

The median TEQ score of 49.0 in our study is indicative of a high level of empathy. This finding is comparable to the results from similar populations that used the same instrument. The Romanian dental, medical, and nursing students recorded median scores of 48.0, 49.0, and 49.1, respectively; the Turkish counterparts scored 52.8 while Australian chiropractic students scored 48.2 [38–41]. On the contrary, findings from Asian medical students showed comparatively lower TEQ scores. The median for China was 42.28 and Korea 44.58 [42, 43]. These stark differences could be indicative of the influence of culture, societal norms, and access to dental care on empathy. These reasons could also explain the statistically significant difference in empathy between Norway, South Africa, and Namibia.

In the present study, a statistically significant difference between the three countries was found, where Norway had the highest empathy scores. The difference in dental empathy in undergraduate oral healthcare students could be due to cultural differences, societal norms, access to dental care, and exposure to oral healthcare from childhood. Additionally, managing the high burden of communicable and life-threatening diseases such as HIV/AIDS and Tuberculosis in South Africa infers that affected societies and health care workers could develop lesser empathy to such health conditions. These attitudes could permeate into other areas of social practice and possibly into the student learning context as well. It points to the deep societal scars that have inevitably formed as a result of prolonged exposure to death and suffering [44]. Furthermore, with the burden of dental caries in South Africa being very high, the dental management is more curative rather than preventative [45, 46]. The dental management in a country with a low burden of dental disease such as Norway [47], which is more preventative than curative, could have a positive impact on the dental anxiety levels. Even though the level of dental anxiety was significantly different between the countries, the mean levels of MDAS (9.51) were lower than what was found in a large study from 2013 in the British population, where the overall mean MDAS score was 10.65 [48]. For the age groups 16–24 years and 25–34 years, the mean score was 11.76 and 11.72, respectively, in the British study, which was close to what was found in Namibia (11.8), the highest in the present study.

We also sought to determine whether there was a relationship between dental empathy and dental anxiety in undergraduate oral healthcare students. In a review by Nair et al., it was found significant relations between anxiety and empathy, and that the relations did not differ across types of anxiety [49]. Moreover, Valdes-Stauber et al. found a strong correlation between dental anxiety and other types of anxiety [50]. Thus, we would assume a relationship between dental anxiety and empathy. However, in our study, there was no relationship between dental empathy and dental anxiety in undergraduate oral healthcare students. One possible explanation could be that dental school creates awareness and can desensitize the students, and they can develop empathy regardless of their dental anxiety levels.

The lack of association could further suggest that the undergraduate dental curriculum sufficiently trains the undergraduate oral healthcare students to be a more empathetic student regardless of their own dental anxiety. This finding could have further implications for ongoing development in the dental curricula aimed at improving dental empathy skills in undergraduate oral healthcare students. The importance of incorporating dental empathy skills training in undergraduate oral health care students is essential to build a well-rounded set of competencies that transcends the technical proficiencies in oral healthcare training.

### Limitations

This was a cross-sectional study design, which is prone to biases like non-response bias and was reliant on self-reported measures. Biases such as social desirability bias where participants could underreport dental anxiety and over-report dental empathy in aligning with being a future healthcare practitioner. Social desirability bias may have had an impact on the study’s results. For instance, if only the less empathetic students did not answer, we could get the impression that the students were more empathetic than they are. However, analyzing the results, we find that there is a considerable variation in the answers, where the median TEQ is 49 (IQR 43.5–52.3). This range implies that students with both high and low empathy answered the questionnaire.

In addition, the overall response rate was low, varying between the different universities. A low response rate may lead to skewed and biased results. A possible explanation for the low response rate is the abundance of surveys taking place. During the COVID pandemic, most primary research was halted. Questionnaires became more common during this time. Students might have suffered from participant fatigue and did not seem keen to respond to this survey. Personalized reminders based on the identification of non-responders were considered non-ethical due to POPI Act in South Africa and Namibia (popia.co.za). We did extend the data collection period for all the schools, and a general reminder was sent to all. Unfortunately, these measures did not increase the response rate. However, there was a difference seen between the universities. In one of the schools, where students themselves were responsible for collecting answers, peers were motivated to respond, and the response rate was higher. Unfortunately, this was not possible at all the schools. However, the average response rate for online surveys varies, and according to a meta-analysis, the average response rate for online surveys was 44.1% [51]. It has also been questioned whether a high response rate necessarily gives more trustworthy results [51, 52]. In a study on surveys among college students, Fosnacht et al. found that surveys with a high response rate were not less prone to bias than surveys with low response rates and concluded that researchers should not worry about low response rates [53]. According to the study, as long as the number of respondents was more than 50, a response rate of 10–15% would give reliable results, and increasing the response rate up to 75% would not reduce response bias considerably [53]. In our study, A power analysis was conducted using 289 participants and 16.0% response rate, and a power of 70.2% was detected, which is below the commonly used threshold of 80%, indicating a risk of Type II error (failing to detect a true difference if it exists). There is also a risk of low external validity, as the results may not be generalizable to the dental students across the different countries. We should be careful when interpreting these results as non-significant findings could be due to the lack of power rather than the lack of existing effect. However, since the number of participants in our study is as high as 298, well above the number required by Fosnacht et al., we are still reasonably confident that our results can be trusted [53] and can clearly articulate that oral healthcare students have lower dental anxiety and higher empathy than other students. We are therefore able to extrapolate that given the nature of dental instruction and programs that oral healthcare students are attuned to develop empathy and become less anxious about dental procedures.

## Conclusion

Oral healthcare students in Africa and Norway showed high empathy levels and low dental anxiety scores. There was no relationship between empathy and dental anxiety in undergraduate oral healthcare students.

## Practical implications for dental education

It is imperative for dental schools to graduate students who are empathetic with attenuated dental anxiety. In so doing, the future practitioner will be more considerate of patients’ views and aspiration, thereby providing quality oral health care for citizens. Dental schools must develop modules, and recruit faculty who can model professional conduct aimed at teaching and developing affective skills, including empathy. Hence, while developing a curriculum, these values should be put into consideration to accommodate students from different backgrounds. Core modules on dental anxiety and empathy in the pre-clinical years must be tailor-made to impart knowledge and soft skills to the students before they start clinical training.
